# Developing a Multiprofessional Mobile App to Enhance Health Habits in Older Adults: User-Centered Approach

**DOI:** 10.2196/54214

**Published:** 2024-04-15

**Authors:** Andressa Crystine da Silva Sobrinho, Grace Angelica de Oliveira Gomes, Carlos Roberto Bueno Júnior

**Affiliations:** 1 Faculty of Medicine of the University of São Paulo Ribeirão Preto Brazil; 2 Department of Gerontology at the Federal University of São Carlos São Carlos Brazil

**Keywords:** information and communications technologies, ICTs, health care, digital inclusion, focus groups, health promotion, user, usability, health literacy, digital competencies, digital skills, mobile phone

## Abstract

**Background:**

Although comprehensive lifestyle habits are crucial for healthy aging, their adherence tends to decline as individuals grow older. Sustaining a healthy life over time poses a motivational challenge. Some digital tools, such as smartphone apps aimed at promoting healthy habits, have been used to counteract this decline. However, a more profound investigation is necessary into the diverse experiences of users, particularly when it concerns older adults or those who are unfamiliar with information and communications technologies.

**Objective:**

We aimed to develop a mobile app focused on promoting the health of older adults based on the principles of software engineering and a user-centered design. The project respected all ethical guidelines and involved the participation of older adults at various stages of the development of the app.

**Methods:**

This study used a mixed methods approach, combining both quantitative and qualitative methodologies for data collection. The study was conducted in Ribeirão Prêto, São Paulo, Brazil, and involved 20 older adults of both genders who were aged ≥60 years and enrolled in the Physical Education Program for the Elderly at the University of São Paulo. The research unfolded in multiple phases, encompassing the development and refinement of the app with active engagement from the participants.

**Results:**

A total of 20 participants used a mobile health app with an average age of 64.8 (SD 2.7) years. Most participants had a high school education, middle-class status, and varying health literacy (mean score 73.55, SD 26.70). Overall, 90% (18/20) of the participants owned smartphones. However, 20% (4/20) of the participants faced installation challenges and 30% (6/20) struggled with web-based searches. The focus groups assessed app usability and satisfaction. Adjustments increased satisfaction scores significantly (Suitability Assessment of Materials: 34.89% to 70.65%; System Usability Scale: 71.23 to 87.14). Participant feedback emphasized font size, navigation, visual feedback, and personalization, and suggestions included health device integration, social interaction, and in-app communication support.

**Conclusions:**

This study contributes to the development of health care technologies tailored to the older adult population, considering their specific needs. It is anticipated that the resulting app will serve as a valuable tool for promoting healthy habits and enhancing the quality of life for older adults.

## Introduction

The older adult population in Brazil is continuously growing [1]. As this demographic expands, critical questions arise regarding how to support older adults in their quest for independence and vitality during the aging process. Furthermore, it becomes essential to identify effective strategies to promote health and improve the quality of life for this segment of society [2].

In the contemporary scenario, marked by constant technological changes, digital products have become an integral part of the context and social life [3,4]. Therefore, there is an urgent need to develop mechanisms that not only enable older adults to comprehend these tools and their potential but also enable older adults to have access to digital products that cater to their specific needs. As Mansell and Tremblay [5] pointed out, knowledge plays a fundamental role in achieving social, economic, and cultural goals, being essential for cultural integration, political participation, and market inclusion.

In this context, information and communications technologies have emerged as vital instruments for developing digital tools that offer numerous benefits to society while contributing to the autonomy and well-being of older adults [6]. These benefits span from the realm of entertainment to the professional sphere and, critically, the field of health, where technology can enhance the well-being of older adults, improve their quality of life, and keep them healthier [7,8].

Tidd and Bessant [8] emphasized that innovation not only is limited to opening new markets but also involves creating meaning and developing new ways of serving. To achieve this goal, it is fundamental to engage all stakeholders in the knowledge-building process, promoting the dissemination of information through interactions such as initiatives coordinated by coalitions of interested professionals and research projects that consider local opinions and choices [5].

Involving the end user is a crucial goal during the development of innovative solutions, not only for evaluation but also in co-design, following a user-centered strategy. Indeed, it is a significant asset of research to base the work on a user-centered approach because it allows building a platform that will address the real needs of the users [3,4].

Digital inclusion emerges as the primary gateway to the use of information and communications technology, enabling individuals to develop the capacity to seek, understand, and apply information according to their needs, acquiring information literacy and digital literacy [9,10]. However, older adults often face obstacles when dealing with these tools as many of them are developed without considering the limitations of users with limited or no digital knowledge and without addressing age-specific issues [3,4]. This mismatch between the condition of older adults and their effective inclusion in the digital environment demonstrates the need for tools that facilitate learning and interaction.

Therefore, this study aims to address the urgent need for research and the search for solutions that can mitigate existing gaps and effectively bring older adults closer to the technological environment. In this context, the central question to be addressed in this research is how to develop a multiprofessional mobile app aimed at promoting health habits in older adults, integrating the perspective of older adults themselves through focus groups to optimize the design and ensure the effective usability of this technological tool for an older adult population.

## Methods

### Ethical Considerations and Trial Design

This research was conducted by the lead researcher CRBJ (associate professor), GAOG (associate professor and research assistant), and ACSS with a degree in health sciences (Faculdade de Medicina de Ribeirão Preto da Universidade de São Paulo [USP]). This project followed the ethical guidelines established by Resolution 466/2012 of the National Health Council and the basic principles of bioethics. All individuals involved in the research received the informed consent form and were informed of confidentiality, with all information disclosed only in scientific events or academic publications. The protocols for this randomized crossover, triple-blind, and placebo-controlled trial were approved by the Ribeirão Preto School of Physical Education and Sport Research Ethics Committee (Process: 58433922.0.0000.5659, on October 26, 2022).

The study details were registered on The Brazilian Clinical Trials Registry (RBR-6wgkzs8: development of an application to improve health in older people). All participants read and signed an informed consent form with detailed information about the experimental protocols.

### Study Type

This study is part of a larger project and describes one of the initial steps. This study consists of technological production research for the development of a mobile app. The approach involves both quantitative and qualitative aspects, with a cross-sectional design. Data were collected from older adults in 3 focus group meetings. In this case, the specific topic was the design of apps for older adults. Data collected in focus group studies are typically used to gain insight into people’s perspectives and experiences. In this study, the data can be used to inform the design of apps that are easier to use and accessible for older adults.

### Study Location and Period

The study was conducted in the city of Ribeirão Prêto, São Paulo, Brazil, with the participation of older adults recruited from the convenience sample of participants in the Physical Education Program for the Elderly (PEFI) at Escola de Educação Física e Esporte de Ribeirão Preto (EEFERP; EEFERP-USP). This study was conducted between January and July 2023.

### Participants

**Figure 1:**
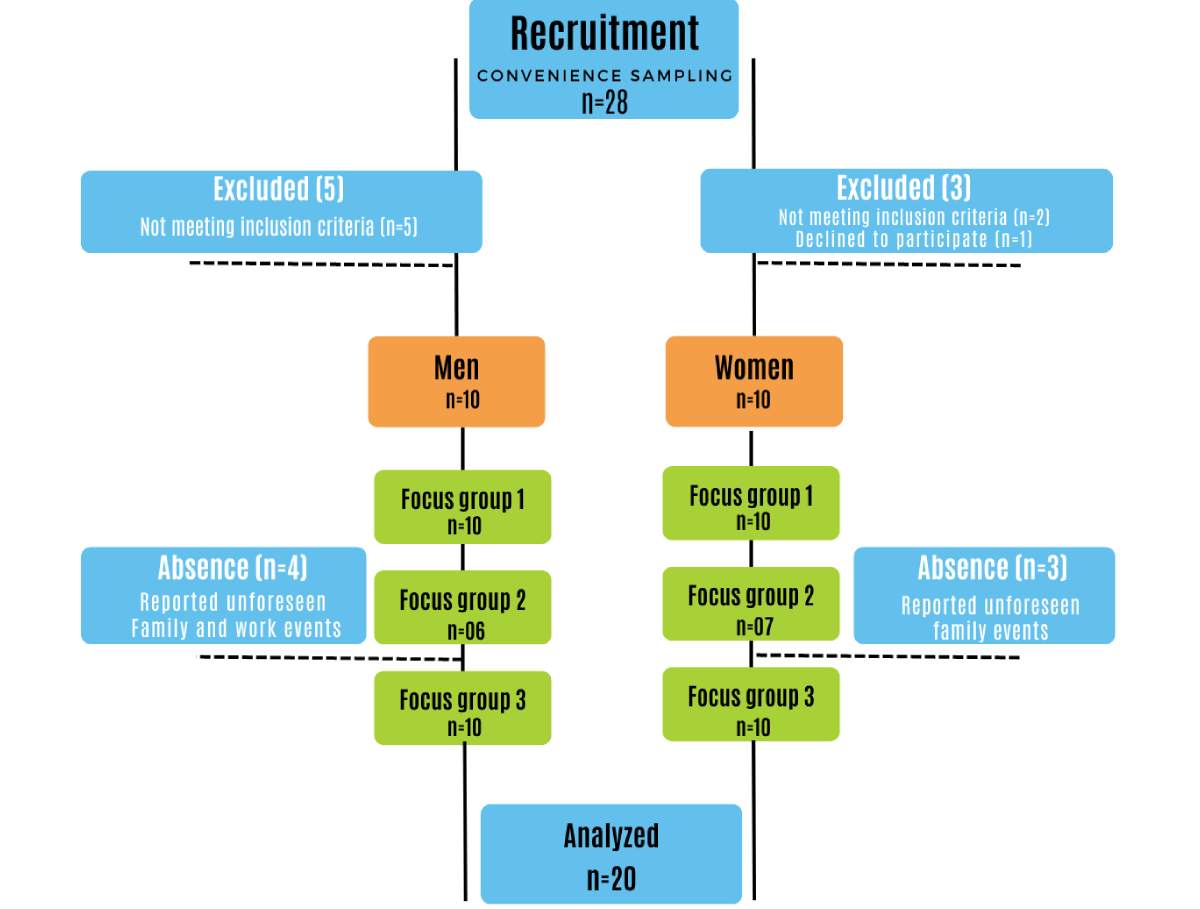
Flowchart for the recruitment of participants.

The study included older adults aged ≥60 years who had participated at some point in the physical training program offered at the EEFERP-USP through the culture and extension project. This PEFI program includes a WhatsApp group that facilitates recruitment through media and technology for all older adults who have already participated in the program. For a focus group testing the app design, it is essential to have a variety of participants with different demographic profiles, interests, and needs [11]. Therefore, for this focus group, we included 10 men and 10 women. A flowchart regarding the recruitment of participants is described in Figure 1.

The inclusion criteria included individuals aged ≥60 years who possessed a mobile device capable of supporting the use of the app and its features—the criterion of having a mobile device is fundamental as the 3 stages of the focus group use this equipment. The exclusion criteria included limitations in performing the assessments outlined in the research, such as visual impairments, hearing impairments, and severe cognitive impairments. The Montreal Cognitive Assessment (MoCA) [12] instrument was used to assess cognitive function and screen for dementia. It was used to analyze whether the difficulty in using the app was related to cognitive performance or the design of the app.

### Research Development

**Figure 2:**
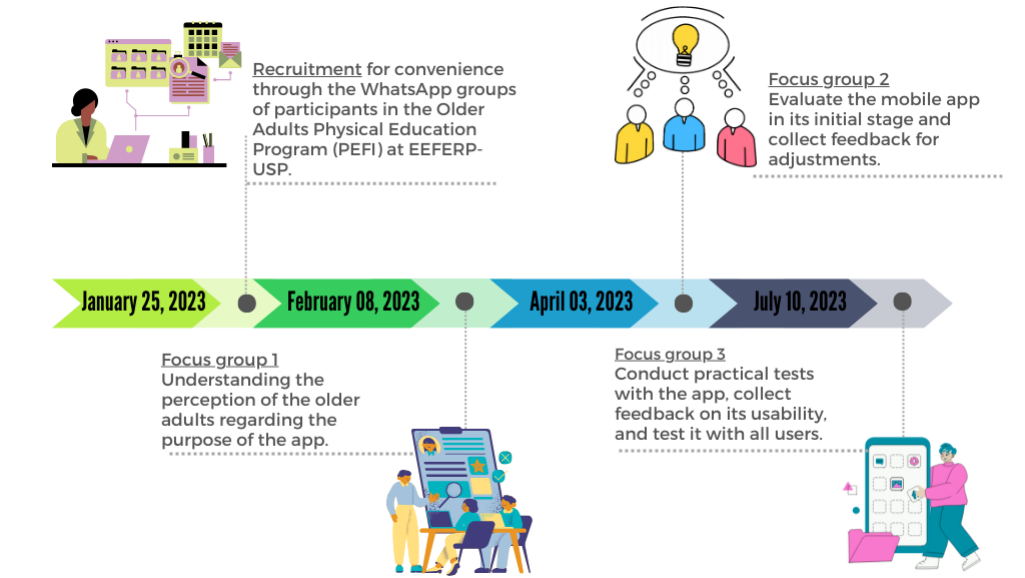
Experimental design. EEFERP-USP: Escola de Educação Física e Esporte de Ribeirão Preto da Universidade de São Paulo.

The project was divided into phases to meet the proposed objectives (Figure 2).

### Development and Adjustment of the App With the Involvement of Older Adults

**Figure 3:**
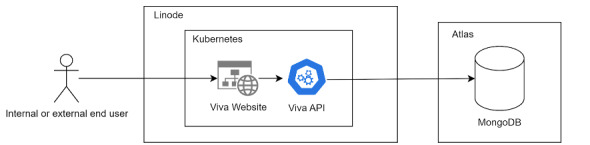
Database diagram. API: application programming interface.

In this phase, the goal was to provide information for the development of a mobile app prototype that tracks and promotes health habits in older adults. Initially, the objectives were established in accordance with the documents and processes recommended by Serviço Brasileiro de Apoio às Micro e Pequenas Empresas for business and technology development. The following documents were created to align project understanding and initiate system modeling:

- Phase 1: business plan: describes the business objectives and the necessary steps to achieve them and identifies the market, product, and existing tools.
- Phase 2: scope: defines the initial project idea and goals and serves as a guide for project production and control.
- Phase 3: Supplier, Input, Process, Output and Customer: a tool from the Six Sigma methodology that manages and optimizes the flow of inputs and products.
- Phase 4: use case diagram: documents the main system functionalities from the user’s perspective.
- Phase 5: flowchart: graphically represents the sequence of screens and their access paths, providing an overall view of the product.
- Phase 6: wireframing: an Agile technique to create a first impression of the project and visualize the layout and flow. We used Figma (Figma, Inc), a cloud-based graphic design and user interface prototyping software.
  After the initial documents were prepared, wireframing was performed using Figma. The Agile methodology was adopted for communication with the lead researcher. Usability testing was conducted with 20 participants aged ≥59 years who were part of the PEFI at the EEFERP-USP. The testing was conducted using the design thinking method, allowing for feedback collection and app adjustments based on user needs.
  The app was developed with Vue and TypeScript for the frontend as a progressive web app and distributed through a Kubernetes cluster with backend communication via an application programming interface developed in Node.js, also using TypeScript. MongoDB was used as the database, hosted within Atlas to facilitate data cross-referencing and subsequent analysis (Figure 3).
  These technologies were chosen for their performance and because they are native web technologies, allowing for broader distribution across various scenarios compared with a more complex framework such as Django, for example. Development time is also crucial, as changes can be made with a short build time afterward.
- Phase 7: execution of focus groups

### Focus Group Objectives

The meetings were conducted at the EEFERP-USP, in a reserved room, a quiet location free of distractions, with audio recording for a duration of 60 to 80 minutes. In addition to the participants, there was a mediator, represented by the lead researcher, and an observer who took notes during the focus groups.

**Figure 4:**
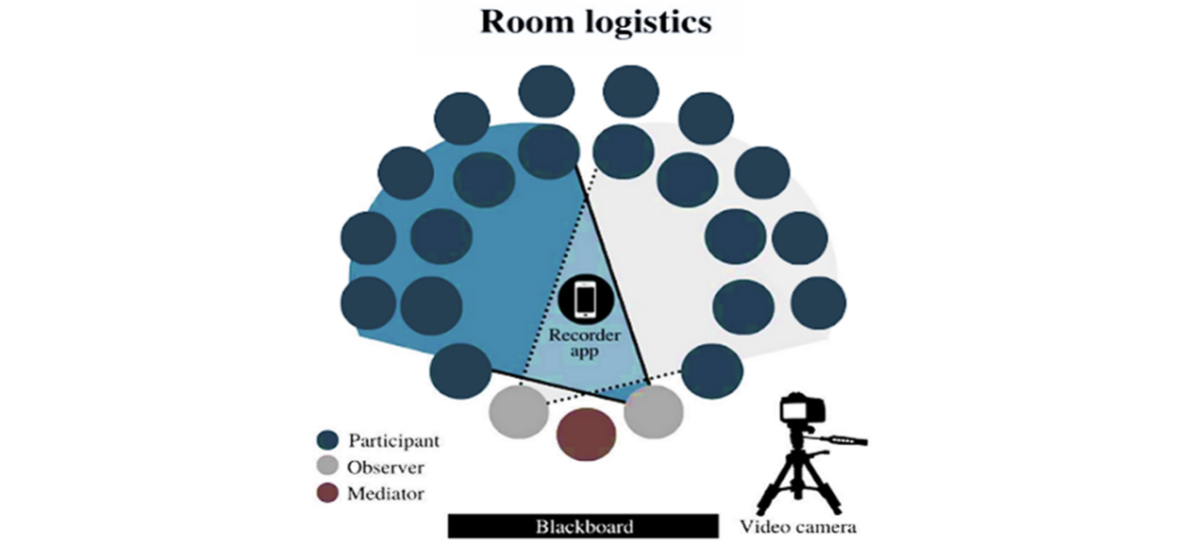
Room layout during the focus group session.

Open-ended questions about the user experience with the system were used during the meetings. In the first and last meetings, the study questionnaires were administered. Closed-ended questions were used in the meetings with caregivers to focus on the system evaluation. Video and audio recordings were used for recording and transcribing the participants’ comments on data security [11]. The layout of the room where the focus group was conducted is illustrated in Figure 4, showing the positions of participants, mediators, and recording equipment.

Focus group 1 comprised a total of 20 participants, equally divided between men and women, with 10 men and 10 women. The primary aim of the first focus group was to comprehend the perceptions of older adults regarding the purpose of the app. The tasks undertaken during this focus group included exploring the health and well-being needs of the older adults, identifying health habits deemed significant by them, gathering suggestions and tips from the older adults for the functionality and practicality of the app, and understanding the expectations of older adults regarding the design and usability of the app.

During the execution of focus group 2, some participants could not attend owing to unforeseen circumstances, medical appointments, and other events, resulting in a total of 13 participants, including 6 men and 7 women. The objective of the second meeting was to assess the early-stage mobile app and gather feedback for adjustments. The tasks carried out during the second focus group meeting included collecting opinions on the appearance, navigability, and features of the app; recording suggestions for improvements and specific adjustments desired by the participants; and exploring the willingness and readiness of older adults to use the app in the future.

In the third focus group, it was possible to bring together all 20 participants with the goal of conducting practical tests with the app, collecting feedback on its usability, and refining the ideal script for future focus group sessions. To achieve this objective, the conduct of the focus group revolved around tasks such as allowing participants to use the app in a controlled environment, documenting observations on how the older adults interacted with the app, gathering feedback on any difficulties or issues encountered during use, and identifying specific aspects of the app that needed improvement.

### Questionnaires Applied

Before the development phase, various questionnaires were applied to gather comprehensive data crucial for the study objectives. These questionnaires covered a range of aspects including participant characterization, cognitive functioning, usability assessment, material suitability, socioeconomic classification, digital competencies, and health literacy.

1. Anamnesis: an initial questionnaire was developed to gather participant characterization for the study. This questionnaire included questions on gender, age, occupation, marital status, profession, and mobile phone brand and model.
2. MoCA: the MoCA is a cognitive screening questionnaire designed to assess cognitive functioning in older adults. The questionnaire consists of 11 items that evaluate attention, memory, reasoning, language, calculation, and orientation. Each MoCA item is scored from 0 to 3, with a maximum score of 30 [12]. A score of ≥26 is considered normal, whereas a score of ≤25 may indicate cognitive decline. However, it is important to note that the MoCA is only a screening test, and a more precise diagnosis should be obtained by consulting a health care professional [12].
3. System Usability Scale (SUS): this is a 10-item questionnaire that measures the usability of a system. It was developed by John Brooke in 1986 [13] and is one of the most widely used usability assessment tools [14]. SUS is a self-administered questionnaire used to evaluate the usability of various systems, including mobile apps, websites, software, and hardware [14]. The questionnaire comprises 10 items that assess system usability on a scale of 1 to 5, with 1 representing “strongly disagree” and 5 representing “strongly agree.” The SUS items evaluate usability in dimensions such as ease of learning, ease of use, efficiency, satisfaction, and memorability [14].
4. Suitability Assessment of Materials (SAM) questionnaire: SAM is a 10-item questionnaire that assesses the suitability of materials for specific applications. It was translated and adapted into Portuguese by Souza et al [15] and is a widely used material assessment tool. SAM is a self-administered questionnaire used to evaluate material suitability for a variety of applications, including medical products, consumer goods, and industrial products. The questionnaire comprises 10 items that assess material suitability on a scale of 1 to 5, with 1 representing “inadequate” and 5 representing “adequate.” SAM items evaluate material suitability based on physical properties, compliance with application requirements, and material safety [15].
5. Brazilian Socioeconomic Classification (CSEB): CSEB is a socioeconomic classification system developed by the Brazilian Institute of Geography and Statistics in 2010 [16]. It is based on a combination of factors, including income, education, and occupation. CSEB is used for research and social planning purposes and is an essential tool for understanding socioeconomic inequalities in Brazil. It can also be used to develop more equitable public policies [16].
6. Digital Competencies and Skills Questionnaire: digital competencies and skills were assessed through a questionnaire created by the researchers based on the Mobile Learning Competence Model for Seniors, specifically designed for older adults [17]. This questionnaire used a 3-point assessment scale: “Yes,” indicating full competence or skill in the evaluated area; “Yes, but with some difficulty,” indicating competence or skill with some challenges; and “No,” indicating the absence of competence or skill in the individual being assessed [18]. The questionnaire covered 6 fundamental categories of digital competencies and skills, including basic technology knowledge, internet navigation skills, mobile app use, digital communication, and digital resource use. The Mobile Learning Competence Model for Seniors provides a solid conceptual framework for measuring the digital competencies of older adults, allowing for a precise and contextualized analysis of their digital skills [17,18].
7. Health Literacy Test (TLS): TLS, validated from the Test of Functional Health Literacy in Adults, was developed by the Federal University of Rio Grande do Sul in 2019 [19]. It is a widely used health literacy assessment tool in Brazil. TLS for Seniors is a 20-item questionnaire that evaluates health literacy in individuals aged ≥60 years [19]. The questionnaire consists of 20 items that assess health literacy on a scale of 0 to 4, with 0 indicating “doesn’t know” and 4 indicating “knows very well.” TLS for Seniors assesses health literacy in dimensions such as understanding medical information, the ability to make health decisions, and the capacity to use health resources effectively [19].

This process will ensure the development of a mobile app that meets the needs and expectations of older adults, promoting greater adherence and effectiveness in health promotion within this population.

### Data Analysis

Data analysis plays a fundamental role in understanding the perceptions, expectations, and needs of older adult participants regarding the mobile app developed in this study. The information collected through specific questionnaires was recorded in a Microsoft Excel software database. Descriptive analyses, such as mean, SD, and percentages, were conducted to characterize the sample in terms of age, gender, education, MoCA, occupational status, marital status, and economic classification. These variables were subjected to a detailed analysis using SPSS statistical software (IBM Corp), with a 2-tailed *t* test to check for potential differences between men and women.

Subsequently, the results of the focus groups were subjected to a qualitative content analysis. The transcriptions of the discussions were coded to identify emerging themes and patterns related to older adult participants’ perception of the app. This qualitative analysis allowed for an in-depth understanding of the participants’ opinions and suggestions presented during the focus groups. Content analysis was carried out according to Bardin [20]. A set of communication analysis techniques was used to obtain, through systematic and objective procedures for describing the content of messages, indicators that allow inferences about the production or reception conditions (inferred variables) of these message points. This analysis was divided into (1) preanalysis, (2) material exploration, and (3) data processing and interpretation.

To validate the questionnaires as a whole, the content validity index was calculated, with the result needing to be >0.90 (>90%) to be considered validated. The purpose was to measure the percentage of older adults who agreed on certain aspects of the instrument and its items. A Likert scale was used with scores ranging from 1 to 4, where 4=entirely appropriate, 3=appropriate, 2=partially appropriate, and 1=inappropriate. The content validity index score was calculated by summing the agreement of the items marked “4” or “3” by 20 older adults.

## Results

### Participant Characterization

In the Results section, we provide a description of our sample based on various key variables. These data are essential for understanding the profile of the study participants. Table 1 displays the means and SDs of age and MoCA scores for both men and women.

**Table 1:** Sample characterization through mean, SD, and percentage of study participants (N=20).

Variables			Men (n=10)		Women (n=10)
Age (y), mean (SD)			64.8 (2.7)		65.1 (4.3)
MoCA^a^ (points), mean (SD)			23.3 (4.4)		23.5 (4.8)
**Education level, n (%)**					
	Elementary school	2 (20)		1 (10)	
	High school	6 (60)		8 (80)	
	Bachelor’s degree	2 (20)		1 (10)	
**Marital status, n (%)**					
	Married	6 (60)		1 (10)	
	Single	2 (20)		2 (20)	
	Divorced	2 (20)		3 (30)	
	Widowed	0 (0)		4 (40)	
**Occupation, n (%)**					
	Retire	4 (40)		0 (0)	
	Salesperson	1 (10)		0 (0)	
	Engineer	1 (10)		0 (0)	
	Mason	1 (10)		0 (0)	
	Electrician	1 (10)		0 (0)	
	Gardener	2 (20)		0 (0)	
	Homemaker	0 (0)		2 (20)	
	Nurse	0 (0)		2 (20)	
	Teacher	0 (0)		2 (20)	
	Secretary	0 (0)		1 (10)	
	Cook	0 (0)		1 (10)	
	Psychologist	0 (0)		1 (10)	
	Receptionist	0 (0)		1 (10)	
**Socioeconomic class, n (%)**					
	Middle class	3 (30)		6 (60)	
	Upper class	4 (40)		2 (20)	
	Lower class	3 (30)		2 (20)	

^a^MoCA: Montreal Cognitive Assessment.

We observed that, men had an average age of 64.8 (SD 2.7) years, whereas women had an average age of 65.1 (SD 4.3) years. The mean scores on the MoCA test were similar for men (mean 23.3, SD 4.4) and women (mean 23.5, SD 4.8). These variables did not show any statistical differences.

Furthermore, we analyzed the distribution of participants according to other important variables. Regarding education, we noticed that most participants (6/10, 60% of men and 8/10, 80% of women) had completed high school. In contrast, a college degree was more common among men (2/10, 20%) than among women (1/10, 10%).

Regarding marital status, 60% (6/10) of the men were married, whereas none of the women in the sample were married. Furthermore, both groups had a similar number of single individuals (2/10, 20%), whereas the number of divorced individuals was higher among women (3/10, 30%) compared with men (2/10, 20%). A significant proportion of women were widowed (4/10, 40%), whereas none of the men in the sample fell into this category.

The occupation of the participants varied, with some groups represented in only one of the genders. For example, all retirees were men, whereas the categories “Homemaker” and the professions such as nurse, teacher, secretary, cook, psychologist, and receptionist were exclusive to women in the sample.

Finally, we explored the socioeconomic class of the participants. The middle-class status was more prevalent among women (6/10, 60%), whereas the upper-class status was more common among men (4/10, 40%). The lower class was relatively uniform among both sexes, with 30% (3/10) of men and 20% (2/10) of women belonging to this category.

These pieces of information provide a comprehensive overview of the demographic and socioeconomic profile of our participants, offering a solid foundation for the analysis and interpretation of the subsequent study results.

### Characterization of Mobile Devices

Participants were also asked to provide the brand and model of their mobile device. There were 4 Samsung smartphones, 2 Motorola smartphones, 2 Redmi smartphones, and 2 iPhones. Of the 20 participants, 18 (90%) reported that the app worked on their mobile models, resulting in a functioning rate of 90% (equivalent to 9 mobile devices). The mobile model that did not work with the app was the Samsung Galaxy A02s model.

### Health Literacy

A variation in scores was observed, ranging from 37 to 98. The average score of the sample was 73.55 (SD 26.70), indicating that most individuals had a good understanding of health information. Some scores stood out, such as 45% (9/20) of the participants who missed only 1 question, achieving 98 points.

- Low health literacy (20-40): Four participants scored in this range, indicating a low level of health understanding, which may affect their ability to make informed decisions about medical care.
- Moderate health literacy (40-60): Four participants scored in this range, suggesting a moderate level of health understanding. They can probably understand basic information, but may have difficulty with more complex material.
- High health literacy (60-100): Twelve participants scored in this range, indicating a high level of health understanding. They are likely to be able to understand and use health information effectively to make informed decisions about their care.

Participant classification was based on the results of the TLS. Of the 20 participants, 12 (60%) were classified as having “adequate” health literacy, with scores above 71; a total of 4 (20%) had “inadequate” health literacy, scoring below 40; and 4 (20%) had “marginal” health literacy, with scores ranging from 40 to 70. This suggests that most participants exhibited an adequate level of health literacy, although there was variation in the results.

### Digital Competencies and Skills

This study addressed various categories related to participants’ knowledge and skills concerning technology and the use of mobile devices. The results provide a comprehensive overview of the technological competencies of the sample, as well as areas where improvements may be considered.

In category 1, which assessed basic technology knowledge, it was observed that all participants owned mobile devices, which is a positive indicator of the penetration of these technologies. However, 10% (2/20) of the participants reported difficulties in turning on and off these devices, suggesting the need for additional guidance in this aspect. In addition, 20% (4/20) of the participants encountered challenges in installing apps, highlighting an area for potential improvement.

In category 2, which investigated internet navigation skills, it was noted that all participants knew how to open a web browser. However, 30% (6/20) of the participants reported difficulties in typing website addresses or conducting web-based searches. Nevertheless, most participants possessed this fundamental knowledge, which was encouraging.

Category 3 addressed the use of mobile apps for learning. Most participants had already used mobile apps to learn something new, demonstrating an interest in leveraging these educational tools. However, 20% (4/20) of the participants had never used them, suggesting an opportunity to promote the use of these resources. Moreover, 40% (8/20) of the participants reported difficulties in searching for and downloading relevant apps, indicating a need for assistance in this area.

In category 4, which analyzed the ability to conduct web-based research, the vast majority of participants had already conducted web-based research to obtain information. However, 30% (6/20) of the participants faced challenges in selecting appropriate keywords, and some struggled to assess the quality of the information found. This underscores the importance of promoting effective search skills and critical content evaluation.

In category 5, which explored digital communication, most participants used emails and instant messaging apps. However, 30% (6/20) of the participants encountered difficulties with messaging apps, indicating an area where training could be beneficial. In addition, most participants demonstrated an awareness of proper digital communication etiquette, which is essential for effective web-based interaction.

Finally, in category 6, which assessed the use of digital resources, most participants exhibited satisfactory skills in handling cameras and media sharing features. Nevertheless, some researchers have encountered challenges in attaching images, photos, and videos in digital communications.

The results of this study indicate that participants have a basic understanding of technology and a general willingness to adopt mobile devices and apps in their lives. However, areas were identified in which some participants faced challenges and difficulties. These insights are valuable in guiding the development of digital empowerment programs and ongoing education to enhance technological skills and promote digital inclusion.

### Satisfaction and App Usability

Table 2 presents the results obtained at 2 points in the focus group, the first interaction with the app (focus group 1) and after adjustments with the users (focus group 3), regarding 2 evaluation metrics: the SUS Validation Index and the SAM Validation Index. These focus groups were conducted to assess user satisfaction and the usability of specific systems or products.

**Table 2:** App satisfaction and usability evaluation at 2 focus group sessions (n=20)^a^.

	SAM^b^ score, mean (SD)	CVI^c^ SAM (%)	SUS^d^ score, mean (SD)	CVI SUS (%)
Focus group 1	34.89 (5.7)	66.11	71.23 (4.7)	70
Focus group 3	70.65 (13)	89.12	87.14 (5.9)	91.13

^a^The values in the table represent the scores of the participants in 2 different focus group sessions, with higher scores indicating higher satisfaction and usability.

^b^SAM: Suitability Assessment of Materials.

^c^CVI: Content Validity Index.

^d^SUS: System Usability Scale.

In focus group 1, participants had an average of 34.89 (SD 5.7) on the SAM questionnaire, indicating moderate satisfaction with the interface of the systems or products under analysis. Regarding the CVI of the SAM questionnaire, the average was 66.11%, suggesting a reasonable assessment of satisfaction. After adjustment, there was a 35.76 increase in the SAM questionnaire results. Therefore, in focus group 3, the results were substantially superior. The participants achieved an average of 70.65 (SD 13) on the SAM questionnaire, reflecting significant satisfaction with the interface. The CVI of the SAM questionnaire, with an average of 89.12%, indicated high satisfaction.

For the SUS questionnaire, participants obtained an average score of 71.23 (SD 4.7), which is a positive evaluation of usability. The CVI of the SUS questionnaire, with an average of 70%, indicated acceptable usability. These results suggest that participants had a more positive user experience in terms of usability than satisfaction with the interface. In the third focus group, there was a 15.91 increase in the SUS questionnaire, with an average score of 87.14 (SD 5.9), indicating excellent usability, whereas the CVI of the SUS questionnaire, with an average of 91.13%, demonstrated that usability was very satisfactory. These results reflect an improvement in the overall user experience, with positive outcomes in both usability and participant satisfaction in focus group 3.

### Results From the Focus Group: Improvements in the Design of the Health App for Older Adults

During the conduct of the focus groups, with a user-centered approach, older adults were highly engaged in discussing and evaluating the current design of the health app. The aim of these meetings was to gather valuable feedback that allowed us to enhance the user experience and make the app more user-friendly for the older adult population. We present the main findings and adjustment points commented on and documented through audio and video recordings by the focus group participants.

Regarding font size and readability, the following comments were made: “The text is too small. I need my glasses to read it”; “There should be an option to increase the font size”; and “It would be great if the text could be read out loud.”

The need for simplified navigation was addressed with comments such as, “Sometimes, I get lost on the home screen. It should be more straightforward”; “The icons are not self-explanatory. We need hints, maybe without images and just text like WhatsApp”; and “There should be a ‘Back’ button on all screens.”

In terms of visual feedback, the following comments were made: “When we complete a task, it’s difficult to know if it was done correctly; there could be a confirmation message”; “Adding a visual confirmation signal would be helpful”; and “Having a contact with a professional within the app encouraging us to use the system would be a great motivator.”

The personalization of notifications was addressed: “I receive too many notifications. It should be possible to choose which ones to receive” and “It would be nice to set up custom reminders for medications.”

Accessibility was emphasized with comments such as, “Some colors are hard to distinguish for people with weak vision”; “The app should be compatible with screen readers”; and “Bright colors make us more excited to use the app.”

Health measurement integrations touched on points such as “Could it connect automatically to our medical devices?”; “A blood pressure recording feature would be wonderful”; “Is it possible to share health tips on social networks?”; and “Can we present this record to our private doctors?”

Concerning in-app communication support, it was suggested that: “Having a customer support chat for questions would be great”; “Could we share our progress with other users of the same age?”; and “Can we create conversation groups on specific topics, such as cooking and recipes?”

In terms of the simplicity of registration, participants raised the following questions: “The registration process is too long. It should be easier”; “The password is too complicated. It should accept simple passwords”; and “Colors could be more distinct to serve as a guide in the instructions.”

Participants indicated the need for an interactive tutorial with the following remarks: “A step-by-step tutorial would help us navigate better”; “The app should explain its features when we start using it”; and “There could be a video section where I can revisit and see how to use it.” They also discussed points related to social experience, including “Can we share our achievements with our friends?”; “Can we invite our friends to view a map of sports facilities in the city?”; “A rewards system to encourage physical activity would be motivating”; and “Can we create competition groups?”

**Figure 5:**
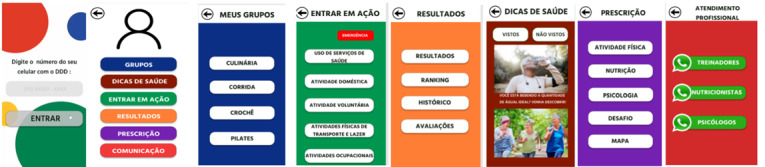
Screen design.

Figure 5 shows the final design of the screens based on the adjustment points mentioned by the focus groups. These points were crucial in improving the health app and making it more accessible and enjoyable for older adults.

## Discussion

### Principal Findings

The main findings of this study include the characterization of its participants, revealing similarities in age and cognitive performance between men and women, but significant differences in education, marital status, and occupation. Furthermore, most participants demonstrated an adequate level of health literacy, albeit with some variability in the results. Regarding digital skills, most older individuals showed basic knowledge of technology, internet navigation skills, and mobile app use, but with some difficulties in certain areas. Finally, the satisfaction and usability of the app significantly improved after adjustments, with older adults highlighting areas for improvement such as font size, simplified navigation, and communication support. These results provide valuable insights for future digital empowerment programs and the development of apps tailored to the older adult population.

### Participant Characteristics

The discussion of the results obtained in our study concerning literature data is essential for contextualizing our findings and assessing how they align or diverge from existing evidence.

Regarding age and scores on the MoCA test, our results indicated that there were no significant differences between men and women. This is in line with several previous studies that did not find consistent differences in cognitive function between the genders [21,22]. However, it is important to note that some studies have reported small differences in certain aspects of cognition, such as verbal memory [23]; however, these discrepancies are not uniform in all studies, as demonstrated by this meta-analysis.

The analysis of education revealed that women in our sample had higher educational levels compared with men, a finding consistent with the literature [24,25]. This may suggest that women in our study had a potential cognitive advantage because of higher education, which should be considered in subsequent analyses.

The discrepancy in marital status is also noteworthy, with only 11% (1/10) married women in our sample, while more than half of the men (6/10, 60%) were married. This may reflect trends broader social contexts, indicating that women are less likely to marry compared to men in some cultures [26,27]. This difference in marital status can influence factors such as social support and support networks, which may have implications for cognitive health.

The occupational differences identified in our sample were also consistent with gender patterns observed in previous studies [28-30]. For example, the exclusivity of certain occupations among women, such as “Homemaker” and some specific professions, reflects gender occupational segregation that persists in many societies. These differences may be related to specific environmental exposures that influence cognitive health.

Regarding the socioeconomic class, the predominance of the upper class among men and the middle class among women is a noteworthy finding that deserves attention. Previous studies have suggested that socioeconomic status can influence cognitive health, with cognitive advantages associated with higher levels of income and education [31]. Therefore, this disparity in socioeconomic class may be an important factor to consider when interpreting our results.

### Characterization of Mobile Devices

The analysis of mobile devices used by the participants revealed a significant presence of mobile phone models from Samsung, Motorola, Redmi, and iPhone, reflecting the diversity of choices available in the current mobile device market [31-33]. This finding is consistent with the current technological reality in which consumers have a wide range of device options to meet their individual needs.

The most notable result was that 90% (18/20) of the participants reported that the app worked smoothly on their devices. This is a positive indicator of the effectiveness of app development for multiple platforms and operating systems, ensuring a satisfactory experience for most users. Such a high level of compatibility is crucial for maximizing the reach and utility of mobile health apps, which often play a significant role in promoting well-being and disease management [34].

The incompatibility detected with Samsung Galaxy A2S models represents a significant issue that deserves attention. Although the overall high rate of app functionality is encouraging, the identification of specific devices that faced problems highlights the complexity of mobile app development. These findings underscore the crucial importance of considering device compatibility during the development process, which is often underestimated but has a substantial impact on user experience [32,33]. Mobile device research and app development have increasingly focused on creating accessible and effective experiences for a wide variety of devices [34]. Variations in hardware, operating systems, and individual settings can create challenges for developers, requiring extensive testing and ongoing optimization to ensure that the app functions reliably in all scenarios. The incompatibility with Samsung Galaxy A2S models highlights the need to consider device and operating system diversity from the outset of the development process [33].

### Health Literacy

The analysis of the HLS results provided an important insight into health literacy among the older adult population. The scores varied considerably, with most participants demonstrating an adequate level of understanding of health information, as evidenced by the average score of 73.55. Furthermore, approximately 45% (9/20) of the participants achieved scores very close to the maximum score, reaching 98 points. These results are in line with a series of previous studies that have also highlighted the relevance of health literacy in the older adult population [35,36]. For example, a systematic review conducted by Berkman et al [35] consistently highlighted the relationship between low health literacy and adverse health outcomes, including lower treatment adherence, inadequate understanding of medical information, and increased health care costs.

Furthermore, classifying participants into health literacy categories (“adequate,” “inadequate,” and “borderline”) underscores the diversity within the older adult population. This variability in health literacy levels has been consistently observed in previous studies [36,37] and reinforces the need for public health approaches that consider the different needs of specific groups based on their health literacy. However, it is important to emphasize that identifying individuals with “inadequate” and “borderline” health literacy underscores the importance of targeted educational strategies and specific interventions for these groups.

Health literacy promotion programs have been shown to be effective in previous studies [38], highlighting the ability to improve the understanding of health information and, consequently, informed decision-making about health among those with low health literacy.

### Digital Competencies and Skills

The assessment of digital competencies and skills of the participants in this study revealed a comprehensive picture of how the investigated population deals with digital technology in various categories. In general, most participants demonstrated a reasonable level of digital knowledge and skills, especially when it comes to basic tasks such as turning on and off mobile devices, opening internet browsers, and sending emails. These findings are in line with the increasing penetration of digital technology into contemporary society [39].

However, areas where some participants faced challenges were also identified, such as selecting appropriate keywords for web-based searches, evaluating the quality of information found on the internet, and the effective use of instant messaging apps. These findings highlight the importance of personalized educational approaches to improve digital skills for specific groups, focusing on the areas where they face difficulties [40].

The results of this study will have significant implications for the development of digital empowerment programs and lifelong education. The ability to use digital technology is essential in an increasingly digitized world, especially for accessing health information, education, and communication. Therefore, investing in initiatives that promote digital inclusion and improve digital skills is crucial to ensure that all individuals, regardless of their age or background, can effectively harness the benefits of digital technology [41].

In summary, this study provides a comprehensive insight into the digital competencies and skills of a group of participants and emphasizes the importance of targeted educational strategies to improve these skills, thus promoting digital inclusion and full participation in the digital society.

### Satisfaction and Usability Assessment

The evaluation of app satisfaction and usability through focus groups provided significant insights into the user experience, and comparing these results with previous studies in the literature enriches our understanding.

Initially, in focus group 1, participants showed moderate satisfaction with the app interface, reflected in an average SAM questionnaire score of 34.89. This initial result is similar to those of studies that have highlighted that satisfaction with app interfaces can vary significantly depending on usability and alignment with user preferences [42].

However, the process of adjusting and refining the app, reflected in focus group 3, had a substantial positive impact on user experience. The participants reported considerable satisfaction, with an average SAM questionnaire score of 70.65, indicating that the implemented improvements aligned the interface with user expectations. This improvement in satisfaction is consistent with the findings of studies that emphasized the importance of adaptability and user-centered design in promoting user satisfaction [43].

Regarding usability, the initial results in focus group 1 already indicated a positive evaluation, with an average SUS questionnaire score of 71.23. This initial result suggests that the usability of the app was in line with previous studies that emphasized the importance of usability in effectiveness and user satisfaction [44]. However, after the app’s improvements and the subsequent assessment in focus group 3, there was a significant improvement in usability. The average SUS questionnaire score increased to 87.14, reflecting excellent usability. This increase in usability is supported by research emphasizing that improvements in the interface, task simplification, and problem-solving can lead to enhanced usability [45].

In summary, the results of this study and comparisons with the literature emphasize the importance of involving users in the development and refinement of digital systems. This not only improves user satisfaction but also enhances usability, promoting a more positive and effective user experience.

### Results From the Focus Groups: Improvements in the Design of the Older Adults Health App

Collecting feedback from the older adults through focus groups played an essential role in identifying areas for improvement in the design of the health app, thereby demonstrating the value of a user-centered approach. The participants highlighted several critical issues that directly affect usability and the user experience.

Regarding readability, concerns about font size and the need for an option to increase it reflect the importance of accessibility for older adults, especially those with vision problems. This is in line with studies that emphasized the need for designs adapted to the specific needs of older adults and bodily changes that occur with aging, such as visual decline [46].

Simplifying navigation was another significant concern, with participants emphasizing the need for a more intuitive interface and self-explanatory icons. This issue is consistent with the literature, which emphasizes the importance of clarity and simplicity in designing apps for older adults—a more straightforward design had better acceptance and adherence in this population [47].

The need for visual feedback to confirm task completion suggests a concern with the effectiveness of actions taken within the app, which can boost user confidence. This aspect is supported by studies highlighting the importance of feedback in user motivation and engagement, showing that it enhances the user’s bond and credibility with the design’s goal [48]. Customization of notifications and integration with medical devices indicate the desire of older adults for a more adaptable and relevant experience. This customization is an important element for improving usability and app acceptance [49].

Accessibility, including compatibility with screen readers and the use of vivid colors, is crucial to meet the needs of older adults with varying levels of visual abilities. This aligns with inclusive design guidelines [50]. The suggestion of communication support and social features reflects the desire of older adults to connect and share experiences with their peers. This social dimension can significantly enhance user engagement and satisfaction [51]. Simplicity in the registration process and the availability of an interactive tutorial are also essential elements to ensure that older adults can make the most of the app. Simplifying the registration process is in line with the literature that emphasizes the importance of minimizing entry barriers and developing fewer back-and-forth paths within screens [52]. Finally, the emphasis on rewards and competitions suggests that gamification elements can be motivating for older adults. This aligns with research demonstrating the benefits of gamification in user motivation and engagement [53].

In summary, the integration of feedback from older adults into the health app design process resulted in a series of improvements that met the needs and preferences of this population. These findings underscore the importance of involving end users, especially the older adults, in the creation of user-centered apps, ensuring a more accessible, user-friendly, and satisfying experience.

### Implications for Public Health

The results of this study have significant implications for public health, especially in the context of an aging population and the growing importance of digital technology in delivering health information and services. First, the analysis of participant characteristics highlighted the importance of considering factors such as gender, education, marital status, occupation, and socioeconomic status when developing health promotion strategies. These variables directly influence cognitive health and the ability to use digital technology.

The assessment of digital skills revealed areas where participants can benefit from additional training and empowerment, especially in more advanced tasks such as evaluating web-based information. Therefore, digital literacy programs targeted at older adults can play a crucial role in empowering these individuals to make the most of digital technologies in health and communication.

Furthermore, the assessment of satisfaction and usability emphasized the importance of designing health apps in a user-centered manner based on user feedback. Continuous improvement of the user interface and usability is essential to ensure that apps effectively meet the needs of older adults.

Finally, the broader implications of this study extend to digital health promotion and the development of digital communication strategies targeting older adults. The increasing digitalization of health information and services requires approaches that ensure equitable access to and proper understanding of health information by older adults, considering their diversity in terms of digital skills and health literacy.

### Limitations and Suggestions for Future Research

Despite providing valuable insights into the interaction of older adults with health apps, this study has limitations that require consideration. First, the sample used in this study was relatively small, which may compromise the generalizability of the results. Efforts to include a more substantial number of participants could improve the representativeness of this research. Another limitation of this study is related to the inherent selection bias in the sample, which was composed of participants already predisposed to using health apps. This may limit the applicability of the conclusions to a broader population of older individuals who may not have the same degree of familiarity or willingness to adopt such technologies.

In addition, it is worth noting that much of the data collected relied on self-reports from participants, which may have introduced memory bias and inaccuracies in responses. Obtaining objective data, supplemented by external assessments, could strengthen the validity of the findings.

In terms of suggestions for future research, we consider some promising directions. First, the inclusion of more diverse samples, covering different ethnic groups, geographic locations, and socioeconomic characteristics, would allow for a more comprehensive understanding of the interaction dynamics between older adults and health apps.

Comparatively, another promising international research suggestion would be the evaluation of the health literacy questionnaire and digital skills of older adults in different countries and cultural contexts, thus providing insights into the contextual determinants of these variables. Furthermore, future studies can explore the health impacts associated with the use of health apps by older adults, including improvements in the self-management of chronic conditions and quality of life. Specifically, the analysis of specific health apps and their features can reveal detailed information about the design and functionality factors that best meet the needs and preferences of older adults.

### Conclusions

In this comprehensive study, we thoroughly examined participant characteristics, mobile device compatibility, health literacy, digital skills, and user satisfaction with a health app designed for older adults. The findings highlight the complexity of the interactions among these factors and underscore the importance of a user-centered approach in the development of digital health apps.

The results suggest that considering the individual differences among older adults, such as gender, education, marital status, and socioeconomic class, is essential to meet their health care needs and ensure an effective digital experience. Furthermore, investing in digital empowerment programs and promoting health literacy can enhance the ability of older adults to use digital technology effectively for accessing health information and services.

The evaluation of user satisfaction and usability of the app emphasized the importance of listening to users and implementing continuous improvements in the user interface. This is crucial to ensure that digital health apps are well received and effective in promoting health and supporting older adults in their health care needs.

Ultimately, this study contributes to the understanding of the intersection between health, technology, and aging, providing valuable insights to inform digital health promotion strategies targeted at this increasingly important population.

## Abbreviations

CSEB: Brazilian Socioeconomic Classification
EEFERP: Escola de Educação Física e Esporte de Ribeirão Preto
MoCA: Montreal Cognitive Assessment
PEFI: Physical Education Program for the Elderly
SAM: Suitability Assessment of Materials
SUS: System Usability Scale
TLS: Health Literacy Test
USP: Universidade de São Paulo
